# Regional lean mass, bone mineral measures, and estimated leg press 1-RM in resistance-trained males

**DOI:** 10.1080/15502783.2026.2746457

**Published:** 2026-10-11

**Authors:** Reza Bagheri, Hanieh Gholamalizadeh, Richard B. Kreider, Mehdi Kargarfard

**Affiliations:** a Department of Exercise Physiology, University of Isfahan, Isfahan, Iran; b International UNESCO Center for Health-Related Basic Sciences and Human Nutrition, Mashhad University of Medical Sciences, Mashhad, Iran; c Faculty of Medicine, Mashhad University of Medical Sciences, Mashhad, Iran; d Exercise & Sport Nutrition Lab, Department of Kinesiology and Sport Management, Texas A&M University, College Station, TX, USA

**Keywords:** Bone mineral content, dual-energy X-ray absorptiometry, estimated one repetition maximum, lean mass, resistance training, body size scaling

## Abstract

**Background:**

Regional lean mass, bone mineral content (BMC), and strength are related in young men, but it is less clear how much these relationships simply reflect shared body and skeletal size in resistance-trained individuals. We therefore examined broad regional associations between lean mass and bone measures, together with selected bone and function relationships, with particular attention to body size.

**Methods:**

This cross-sectional secondary analysis used baseline data from 44 resistance-trained males from a previous four group trial. Whole body dual energy X ray absorptiometry (DXA) provided broad upper body and lower body lean mass measures, BMC, projected bone area, and areal bone mineral density (BMD). We examined associations using Pearson correlations and linear regression with HC3 robust standard errors. Models adjusted for age and height were considered alongside body mass, projected area, and allometric models. Benjamini Hochberg correction across all 18 adjusted cross-sectional tests was prioritized.

**Results:**

Regional lean mass was positively associated with several BMC outcomes in models adjusted for age and height, including arm BMC (standardized *β* = 0.605), rib BMC (*β* = 0.608), leg BMC (*β* = 0.429), and pelvis BMC (*β* = 0.550). Several estimates became smaller or less precise when body mass, projected bone area, or allometric scaling was considered. Pelvis BMC was conditionally associated with estimated leg press 1-RM after adjustment for age, height, and lower body lean mass (*β* = 0.501; 18 test BH q = 0.001). This association, however, cannot distinguish the influence of skeletal size, body proportions, training history, or other unmeasured factors.

**Conclusions:**

In resistance-trained males, regional DXA lean mass and bone measures appear to form a phenotype that is strongly influenced by body size. Associations from models adjusted for age and height should therefore be interpreted alongside the size conditioned analyzes. The association between pelvis BMC and estimated leg press 1-RM is best viewed as an exploratory conditional finding within this broader phenotype, rather than evidence of a distinct skeletal contribution to strength.

## Graphical abstract

**Figure:**
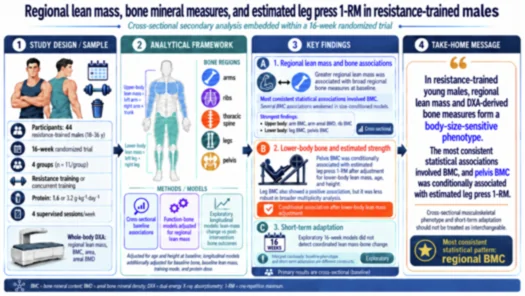

## 1. Introduction

Skeletal muscle and bone are usually measured as separate compartments, yet in practice they function together as a system that bears and transmits force. Muscle contractions account for much of the dynamic loading experienced by the skeleton, while bone geometry and joint architecture shape how force is transferred through the limbs and axial skeleton. Both tissues are also influenced by body size, habitual activity, nutrition, endocrine factors, and genetic and developmental history, and they communicate through local and systemic signaling pathways [1–3]. Regional muscle mass may therefore reflect both current force-generating capacity and accumulated loading exposure, whereas regional bone characteristics may reflect structural demands experienced during growth and training. These shared influences make regional associations plausible, but they also make causal interpretation difficult.

How mechanical loading is distributed across the body is central to this question. Pressing and pulling exercises, lower body resistance exercise, jumping, and cycling do not load every skeletal region in the same way [4]. Muscle attachment sites, joint geometry, technique, external resistance, and movement velocity all influence the direction and magnitude of strain. Whole body measures may therefore obscure relationships that are easier to see when upper body and lower body tissues are considered separately. Previous work in young men has reported positive relationships among regional lean mass, muscular strength, and bone outcomes, although their magnitude varies by anatomical region and by the covariates included [5–7].

An important point when interpreting these data is that bone mineral content (BMC) and dual-energy X-ray absorptiometry (DXA)-derived areal bone mineral density (BMD) do not represent the same property. BMC describes mineral quantity within a projected region and consequently retains substantial information about skeletal size. Areal BMD divides mineral content by two-dimensional projected area, but it is not a volumetric density and remains sensitive to bone size, positioning, and region-of-interest definition [8]. A stronger association with BMC could therefore reflect a biologically meaningful loading phenotype, shared body or skeletal size, or both. Examining BMC and areal BMD in parallel, and considering models that include body mass or projected bone area, helps to distinguish these interpretations without assuming that one metric is intrinsically superior.

Physical function adds another perspective because muscle quantity alone does not determine strength or power [9]. Neural activation, muscle architecture, moment arms, limb proportions, technique, training history, and joint geometry also matter [10,11]. Regional bone measures may capture aspects of skeletal dimensions or accumulated high load exposure that are not represented fully by DXA-derived lean mass [2,6]. Thus, an association observed after adjustment for regional lean mass would indicate only that the measured covariates did not fully account for the cross-sectional relationship; it would not identify a distinct skeletal contribution to force production.

Much of the research on muscle and bone relationships has focused on children, older adults, clinical populations, or people with little resistance training experience [3,12,13]. Young adults with substantial resistance training experience represent a distinct physiological phenotype. Progressive overload repeatedly exposes the musculoskeletal system to high muscle forces, alters the regional distribution of lean tissue, and may preserve or augment skeletal characteristics at loaded sites [4,10]. Physically active young men nevertheless show substantial variation in bone mass and size related to body size and exercise exposure [6], and observations in collegiate athletes suggest that regional bone mineral can continue to change during early adulthood under sport-specific loading [14]. Evidence that brings regional lean mass, regional bone measures, and physical function together in resistance-trained adults remains limited.

Previous studies have shown that regional lean mass, bone mineral measures, and strength are related in young men [5–7]. The unresolved question is how much of these relationships persists once overall body size and skeletal size are considered in men who are already adapted to regular resistance training [5–7]. This population is informative because regular resistance training exposes the musculoskeletal system to repeated high force loading and produces adaptations in muscle size and strength [4,10]. Within such a relatively trained phenotype, variation in regional lean mass, skeletal characteristics, and performance can therefore be examined with less emphasis on simple trained versus untrained group differences. We therefore focused on how regional DXA relationships changed across different approaches to body size adjustment and whether selected bone and function relationships were still apparent after accounting for the available lean mass and size related covariates.

The same 44 completers contributed to the final analyzes of the three earlier publications [15–17], but the estimands were different (Table 1). The first report tested group-by-time effects of training mode and protein dose on body composition, estimated strength, performance, and liver/kidney markers [15]. The second tested intervention effects on regional bone outcomes [17]. The third related changes in upper- and lower body lean mass to changes in performance outcomes [16]. None of those analyzes tested the present baseline cross-sectional regressions linking regional lean mass to regional bone measures, or the conditional association of regional bone measures with estimated 1-RM after lean mass and size adjustment. Thus, source measurements necessarily overlap because they arose from one cohort, whereas the hypotheses, time scale, estimands, and statistical models of the present paper are distinct. A consolidated comparison of publications and participant flow is provided in Table 1 and online resource Table S1.

**Table 1.** Analytical distinction among publications arising from the parent trial.

Publication	Analysis sample	Primary question	Principal variables	Primary analysis	Information unique relative to current paper
JISSN 2023	48 randomized; 44 final (11/group)	Intervention effects of CT vs RT and 1.6 vs 3.2 g·kg^−1^·day^−1^ protein	Body composition, estimated strength, performance, aerobic capacity, liver/kidney markers	Repeated-measures group × time treatment effects	No baseline regional muscle-bone or conditional bone-function regressions
Nutrients 2024	48 randomized; 44 final (11/group)	Intervention effects of CT/RT and protein dose on regional bone outcomes	Regional area, BMC, and areal BMD	Pre-post group × time treatment effects	No baseline lean mass-bone or lean-adjusted function-bone regressions
Frontiers in Nutrition 2024	48 randomized; 44 final (11/group)	Whether within-person lean-mass changes tracked changes in strength, endurance, and power	Change in upper/lower lean mass and change in performance	Change-change Pearson correlations	No baseline regional bone associations
Current analysis	Same 44 completers	Baseline regional phenotype and size-conditioned function-bone associations	Regional lean mass, BMC/BMD, projected area, estimated 1-RM/performance	Cross-sectional correlations; HC3 regressions; complementary body-size, projected-area, and allometric analyzes; influence and multiplicity robustness analyzes	Unique estimands: baseline regional lean-bone models and conditional bone-function models

All publications derive from the same parent trial and therefore necessarily reuse source measurements. Their hypotheses, time scales, estimands, and statistical models differ. In earlier allocation descriptions, *n* = 12/group denotes the 48 randomized participants; participant-flow/results sections report one withdrawal per group and final analyzes based on 44 completers (*n *= 11/group). CT, concurrent training; RT, resistance training; BMC, bone mineral content; BMD, DXA-derived areal bone mineral density; HC3, heteroskedasticity-consistent covariance estimator 3.

The primary objective was to characterize the cross-sectional relationship between broad upper body and lower body lean mass compartments and regional BMC and areal BMD, while examining how these associations changed after accounting for body size and skeletal size. Models adjusted for age and height describe the broad phenotype, whereas models incorporating body mass, projected area, and allometric scaling provide complementary views of the same relationships and are given equal interpretive weight. Secondary analyzes examined a limited set of predefined regional and performance relationships and were considered hypothesis generating. The exploratory 16 week change analyzes are reported only in the online resource.

## 2. Methods

### 2.1. Study design and participants

This was primarily a cross-sectional analysis of baseline data collected within a previously reported randomized four group, 16-week intervention involving young, healthy, resistance trained males. Forty-eight participants were randomized and four later withdrew, corresponding to 8.3% attrition. One participant withdrew from each group, leaving 44 completers (11 per group). The archived participant level dataset available for this analysis contains these 44 completers; baseline records for the four participants who later withdrew could not be retrieved. The earlier publications do not provide participant level baseline characteristics for those four withdrawals, so the magnitude or direction of any selection bias cannot be estimated retrospectively. The even distribution of withdrawals across groups and the heterogeneous reasons for withdrawal are not suggestive of concentration within a single group, but they do not establish that completers and non-completers were comparable at baseline. The protocol was approved by the Ethics Committee of the University of Isfahan (IR.UI.REC.1400.098), conducted according to the Declaration of Helsinki, and registered prospectively (IRCT20191204045612N2).

Eligibility required at least one year of resistance training experience, habitual resistance training three to four times per week, self-reported sleep of 7–8 h/day, habitual protein intake below approximately 1.6 g·kg^−1^·day^−1^, no musculoskeletal disorder at enrollment, and no anabolic steroid or other illegal muscle enhancing agent use during the preceding year. These criteria, and the intervention that followed, describe the source cohort; they do not make the primary baseline association analysis a randomized comparison.

### 2.2. Intervention

The source cohort came from a four-group trial comparing resistance training (RT) with concurrent training that combined resistance exercise and cycling (CT), each combined with protein targets of 1.6 or 3.2 g·kg^−1^·day^−1^. These post-baseline interventions are described only to establish cohort provenance; they are not exposures in the primary cross-sectional analyzes. Detailed training progression, concurrent training rationale, adherence, and intervention effects have been reported previously [15–17].

### 2.3. Dietary intervention

The parent trial used protein targets of 1.6 or 3.2 g·kg^−1^·day^−1^, supported by post-exercise whey protein and dietary counseling. Full dietary procedures, achieved intakes, and adherence are reported in the parent trial [15].

### 2.4. Anthropometry and dual-energy X-ray absorptiometry

Participants reported after an overnight fast and were instructed to void within 30 min of testing and avoid alcohol, caffeine, and other diuretics before assessment. Body mass and height were measured to the nearest 0.1 kg and 0.1 cm, respectively. Whole-body DXA (Hologic Discovery, Hologic Inc., USA; APEX software version 4.5.3.2) quantified total and regional lean mass, fat mass, body fat percentage, estimated visceral adipose tissue mass, bone area, BMC, and areal BMD. Scans were performed by the same technician using image-comparison mode for serial assessments and manufacturer calibration procedures. Previous laboratory reliability was an intraclass correlation coefficient (ICC) of 0.998 and coefficient of variation (CV) <1% for body fat percentage, and an ICC of 1.00 and CV <1% for lean mass. Visceral adipose tissue was expressed in grams. Regional bone outcomes included the left and right arms, ribs, legs, thoracic spine, lumbar spine, and pelvis.

For this secondary analysis, bilateral arm, rib, and leg BMC were calculated by summing the left and right regions, and bilateral areal BMD was calculated as summed BMC divided by summed projected bone area. Upper body composite lean mass consisted of left arm, right arm, and trunk lean mass, whereas lower body lean mass consisted of both legs. These measures represent broad body compartments rather than anatomically matched muscle and bone units. In particular, the upper body composite includes trunk tissue when it is compared separately with arm, rib, and thoracic spine outcomes, and leg lean mass is not anatomically identical to pelvis BMC. Arm only and trunk only lean mass variables, together with the original scan files needed to reconstruct them, were unavailable. Because lean mass, projected area, BMC, and BMD were derived from the same DXA examination, shared dependence on body size, common regional boundaries, and correlated measurement error may strengthen cross-sectional relationships without demonstrating biological coupling between muscle and bone. Site specific regional scan rescan precision, least significant change values, analyst blinding, and detailed records of regional reanalysis were unavailable. Training age and cumulative loading could be related to both regional lean mass or BMC and estimated strength, while pelvic and limb dimensions could influence both measured BMC and the mechanics of the leg press. Their absence therefore leaves plausible residual confounding in the function models.

### 2.5. Strength, endurance, power, and performance

Maximal strength was not measured directly in this study. All strength outcomes in this manuscript are repetition-derived estimated 1-RM values and therefore include prediction error in addition to equipment specific and technique related variability. Participants were familiar with the testing procedures before baseline assessment. Chest-press testing preceded the plate-loaded leg press and was conducted in the morning. Strength was estimated from the heaviest load and corresponding repetition count using estimated 1-RM = weight/[1.0278 − (0.0278 × repetitions)] [18], with sets limited to 10 repetitions or fewer. After 5 min of recovery, muscular endurance was assessed as the number of technically acceptable repetitions completed at 75% of estimated 1-RM. Upper- and lower body peak power were measured during seated 30-s Wingate tests on a Monark 894e ergometer, using resistance equal to 5.5% and 7.5% of body mass, respectively; peak power was recorded with Monark Anaerobic Test software version 3.3.0.0. Before vertical-jump and pull-up testing, participants completed a 5-min self-selected run or cycle, dynamic movements, 10 push-ups, and 10 bodyweight squats, followed by 2–3 min of rest. The best vertical-jump height and the maximum number of pull-ups were recorded from up to three attempts, with approximately 60–180 s between attempts. Estimated maximal oxygen uptake was obtained with the Ekblom-Bak submaximal cycle test. Further details on equipment and test retest reliability were not available beyond the parent-trial report [15].

The parent protocol further specified a 10-min general/specific warm-up before estimated 1-RM testing, two strength attempts, a maximum of 10 repetitions to fatigue, and 3–5 min of rest between attempts, with no external arousing stimulus. The reported strength values are therefore repetition-derived estimates rather than directly observed maximal lifts. The archived publications and dataset did not provide the leg press manufacturer/model, sled or plate-loaded geometry, seat/back angles, standardized foot position, repetition cadence, exact range-of-motion criterion, or participant level repetition counts used in each estimate, and no test-retest reliability coefficient for the estimated leg press 1-RM was available. Because absolute loads are equipment specific, the values should not be interpreted as directly comparable with those from a different leg press apparatus.

### 2.6. Outcomes and analysis hierarchy

The cross-sectional analysis included 10 models relating lean mass to bone measures and eight models relating bone measures to function. The main estimand was descriptive: the association between broad DXA lean mass compartments and regional bone measures in a resistance-trained sample. Models adjusted for age and height characterize this broad phenotype, while body mass, projected area, and allometric models show how strongly the findings depend on common scaling and are given equal interpretive weight. The pelvis BMC and estimated leg press 1-RM model was one of the predefined bone and function relationships for this secondary analysis, not a prospectively registered primary outcome. Detailed robustness procedures and the exploratory longitudinal analyzes are reported mainly in the online resource.

### 2.7. Statistical analysis

Analyzes included the 44 participants with complete baseline and post intervention data. Continuous variables are reported as mean ± standard deviation (SD). We first inspected distributions and bivariate plots, then evaluated regression assumptions using residual-versus-fitted and normal quantile-quantile plots. Externally studentized residuals, leverage, Cook's distance, and variance-inflation factors (VIFs) were used to identify influential observations and possible collinearity. Regression confidence intervals and *P* values were based on HC3 heteroskedasticity-consistent standard errors. HC3 was used to improve inference under heteroskedasticity [19]; it was not regarded as a solution to omitted-variable bias, measurement error, or instability caused by high-leverage observations. Pearson correlations were the primary unadjusted association measure, with Spearman rank correlations used to assess robustness. Adjusted associations were estimated with ordinary least-squares regression. Continuous outcomes and predictors were standardized before analysis, so a standardized *β* represents the standard-deviation difference in the outcome associated with a 1-SD difference in the predictor, conditional on the other variables in the model. Unstandardized coefficients and 95% confidence intervals (CIs) are also reported to preserve the original measurement units. Primary baseline muscle and bone models included age and height. Because training mode and protein dose were assigned after baseline assessment, they were not included in the primary baseline models. Baseline bone and function models also included the broadly aligned regional lean mass measure. Complementary models added randomized assignment, baseline body mass, or corresponding projected bone area. These models answer different questions. The adjusted for age and height models describe the broader regional phenotype, including some shared variation with body and skeletal size. Body mass adjustment reduces shared variation with overall size, whereas projected area adjustment estimates BMC conditional on regional bone size. Longitudinal models regressed the post intervention bone outcome on change in regional lean mass while adjusting for baseline bone, baseline regional lean mass, training mode, and protein dose. We did not fit group-specific regressions or interaction terms because each randomized group contained only 11 participants.

Covariates were selected to answer different, complementary questions rather than through statistical screening. Age and height were used in the conventional phenotype models because they account for basic demographic and linear body size differences while retaining the overall musculoskeletal phenotype that was the starting point of the analysis. Body mass was then added to examine whether associations persisted after accounting for overall mass related scaling, whereas projected bone area was added to ask whether BMC varied beyond the two-dimensional size of the measured skeletal region. Log allometric models provided a further assessment of proportional scaling. No single specification was treated as uniquely biologically correct. Instead, interpretation was based on how the estimate changed across these models: consistency suggests relative robustness to the measured scaling factors, whereas attenuation indicates that common body or skeletal size contributes materially to the association. Projected area adjustment is conceptually distinct from areal BMD, which is calculated as BMC divided by area, and none of these models isolates a unique skeletal contribution to strength.

Benjamini-Hochberg (BH) false discovery rate adjustment was applied [20] across all 18 adjusted cross-sectional tests and was the principal multiplicity control. Family-specific q values were retained only as secondary descriptive information. Detailed influence, leave-one-out, Huber, bootstrap, variance-inflation, and broader 26-test analyzes are reported in the online resource for reproducibility rather than treated as separate layers of primary inference. Analyzes were conducted in Python using pandas, SciPy, statsmodels, and Matplotlib. With *n* = 44, the analysis had limited ability to detect small partial associations; imprecise or nonsignificant estimates were therefore not interpreted as evidence of equivalence or absence. Correlation matrices and variance inflation diagnostics are provided in online resource Tables S2A-S2C, and the detailed influence and robustness analyzes are reported in online resource Table S3.

## 3. Results

### 3.1. Participants and data completeness

Forty four of the 48 randomized participants completed the intervention and make up the archived participant level dataset used in this analysis (11 per randomized group). One participant withdrew from each group because of lack of time, loss of interest, COVID-19, or musculoskeletal injury. This accounting is consistent with the final participant flow and the *n* = 11 per group results reported in earlier publications from the cohort; references to *n* = 12 per group describe the original allocation of 48 participants. Baseline records for the four participants who later withdrew were not available in the archive and therefore could not be added to the baseline cross-sectional analysis. Table 1 distinguishes the analytical samples and aims across publications, and online resource Table S1 summarizes participant flow. Baseline characteristics of the 44 participants included in the analysis are shown in Table 2.

**Table 2.** Baseline characteristics of the 44 participants included in the cross-sectional analysis, shown by subsequent randomized group.

Variable	CT + 1.6 (*n* = 11)	CT + 3.2 (*n* = 11)	RT + 1.6 (*n* = 11)	RT + 3.2 (*n* = 11)
Age (years)	26.73 ± 6.69	25.64 ± 6.76	26.82 ± 6.49	27.91 ± 5.72
Height (cm)	178.45 ± 5.73	179.82 ± 7.97	180.55 ± 7.85	182.00 ± 6.60
Body mass (kg)	83.80 ± 10.61	81.65 ± 10.68	82.11 ± 9.18	85.22 ± 10.95
Upper-body composite lean mass (kg)	36.37 ± 4.11	35.81 ± 4.01	35.72 ± 3.90	39.61 ± 7.32
Lower-body lean mass (kg)	20.02 ± 2.46	20.37 ± 1.95	20.62 ± 2.93	21.67 ± 4.13
Arm BMC (g)	454.63 ± 61.72	438.87 ± 79.62	430.11 ± 65.07	453.62 ± 100.30
Arm areal BMD (g/cm^2^)	0.96 ± 0.08	0.91 ± 0.08	0.91 ± 0.08	0.92 ± 0.08
Leg BMC (g)	1316.14 ± 181.72	1239.86 ± 170.72	1272.67 ± 251.62	1209.18 ± 224.28
Leg areal BMD (g/cm^2^)	1.60 ± 0.14	1.52 ± 0.13	1.53 ± 0.18	1.46 ± 0.12
Pelvis BMC (g)	360.08 ± 79.65	341.29 ± 44.24	349.19 ± 89.59	322.79 ± 65.11
Pelvis areal BMD (g/cm^2^)	1.35 ± 0.11	1.26 ± 0.13	1.26 ± 0.18	1.21 ± 0.09
Estimated chest press 1-RM (kg)	96.73 ± 21.50	100.82 ± 19.37	98.27 ± 17.73	107.82 ± 16.41
Estimated leg press 1-RM (kg)	412.55 ± 76.78	388.27 ± 73.56	390.36 ± 70.90	408.73 ± 69.53

Data are mean ± SD. Randomized groups are shown only to describe cohort provenance; group assignment occurred after these baseline measurements and is not part of the primary cross-sectional estimand. No baseline significance tests were performed. 1-RM, repetition-derived estimated one-repetition maximum; BMC, bone mineral content; BMD, DXA-derived areal bone mineral density; CT, concurrent training; RT, resistance training; SD, standard deviation.

### 3.2. Unadjusted regional muscle and bone associations

Unadjusted baseline associations are summarized in Table 3. The relationship between upper body composite lean mass and arm BMC is illustrated in Figure 1, while the corresponding lower body relationships with leg BMC and pelvis BMC are shown in Figures 2 and 3, respectively. Upper body composite lean mass correlated with arm BMC (r = 0.780), arm areal BMD (r = 0.649), rib BMC (r = 0.663), and thoracic spine BMC (r = 0.569). Lower body lean mass correlated with leg BMC (r = 0.720) and pelvis BMC (r = 0.586).

**Table 3.** Unadjusted baseline associations among regional lean mass, bone measures, and physical-function outcomes.

Family	Relationship	Pearson r	P	Family BH q	Spearman ρ	P
Upper-body muscle-bone	Arm BMC	0.780	<0.001	<0.001	0.767	<0.001
	Arm areal BMD	0.649	<0.001	<0.001	0.665	<0.001
	Rib BMC	0.663	<0.001	<0.001	0.691	<0.001
	Rib areal BMD	0.367	0.014	0.017	0.425	0.004
	Thoracic-spine BMC	0.569	<0.001	<0.001	0.567	<0.001
	Thoracic-spine areal BMD	0.281	0.065	0.065	0.225	0.142
Lower-body muscle-bone	Leg BMC	0.720	<0.001	<0.001	0.710	<0.001
	Leg areal BMD	0.407	0.006	0.008	0.370	0.014
	Pelvis BMC	0.586	<0.001	<0.001	0.611	<0.001
	Pelvis areal BMD	0.230	0.133	0.133	0.221	0.150
Upper-body function-bone	Estimated chest press 1-RM - arm BMC	0.478	0.001	0.004	0.410	0.006
	Upper-body peak power - arm BMC	0.325	0.031	0.063	0.298	0.049
	Pull-ups - rib areal BMD	−0.038	0.805	0.805	−0.023	0.882
	Estimated chest press 1-RM - thoracic-spine areal BMD	0.189	0.219	0.292	0.128	0.408
Lower-body function-bone	Estimated leg press 1-RM - leg BMC	0.233	0.127	0.170	0.194	0.208
	Lower-body peak power - leg BMC	0.178	0.247	0.247	0.141	0.361
	Vertical-jump height - pelvis areal BMD	0.357	0.017	0.057	0.376	0.012
	Estimated leg press 1-RM - pelvis BMC	0.331	0.028	0.057	0.381	0.011

Pearson r was the primary unadjusted association measure; Spearman *ρ* was a rank-based robustness analysis. Family BH q values are shown for descriptive continuity with the anatomically structured analysis. Primary adjusted inference prioritizes the BH correction across all 18 adjusted cross-sectional tests in Tables 4 and 5. All relationships use *n* = 44. 1-RM, repetition-derived estimated one-repetition maximum; BH, Benjamini-Hochberg; BMC, bone mineral content; BMD, DXA-derived areal bone mineral density.

**Figure 1.**
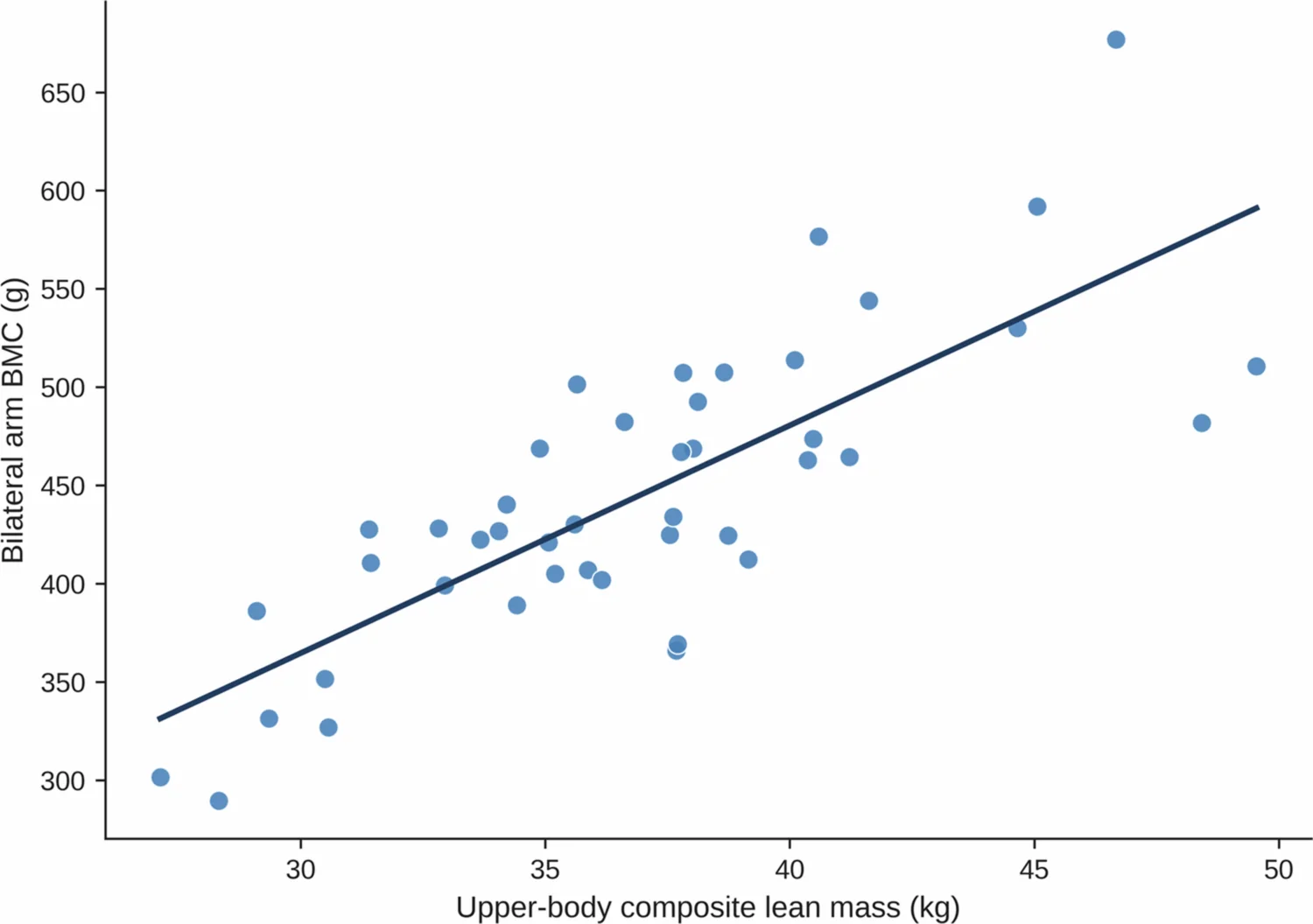
Baseline relationship between upper-body composite lean mass and bilateral arm BMC. Each point represents one of the 44 analyzed participants. Randomized training/protein groups are intentionally not encoded because assignment occurred after baseline and is not part of the cross-sectional estimand. The solid line is the pooled descriptive ordinary least-squares fit. Pearson r = 0.780 (*P* < 0.001). Upper-body composite lean mass combines both arms and trunk and is therefore a broad compartment rather than an anatomically matched exposure. BMC, bone mineral content.

**Figure 2.**
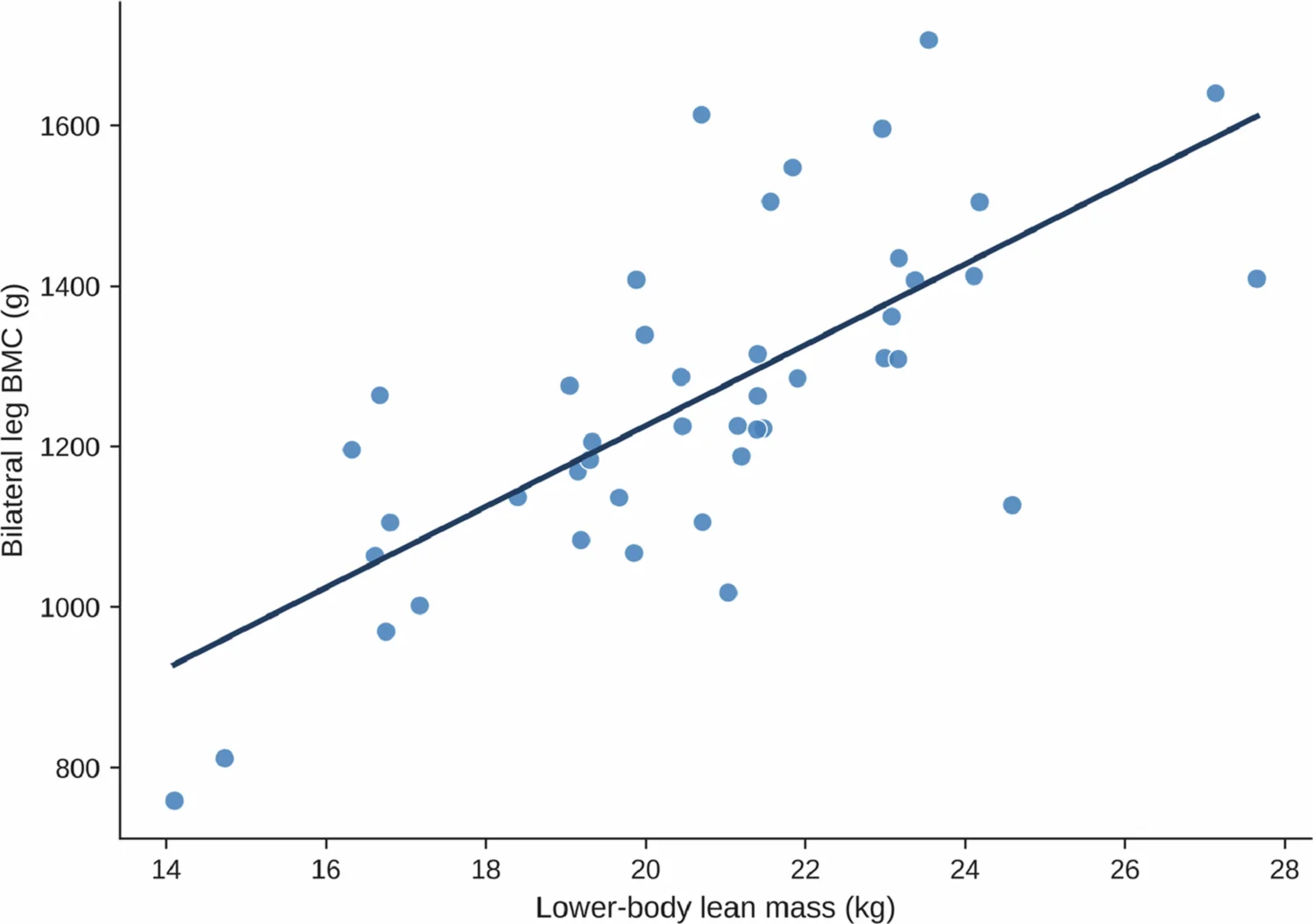
Baseline relationship between lower-body lean mass and bilateral leg BMC. Each point represents one of the 44 analyzed participants; randomized intervention groups are pooled because they were assigned after baseline. The solid line is the pooled descriptive ordinary least-squares fit. Pearson r = 0.720 (*P* < 0.001). Complementary body-mass, projected-area, and allometric analyzes are reported in Table 6 and are given equal interpretive importance when evaluating common scaling. BMC, bone mineral content.

**Figure 3.**
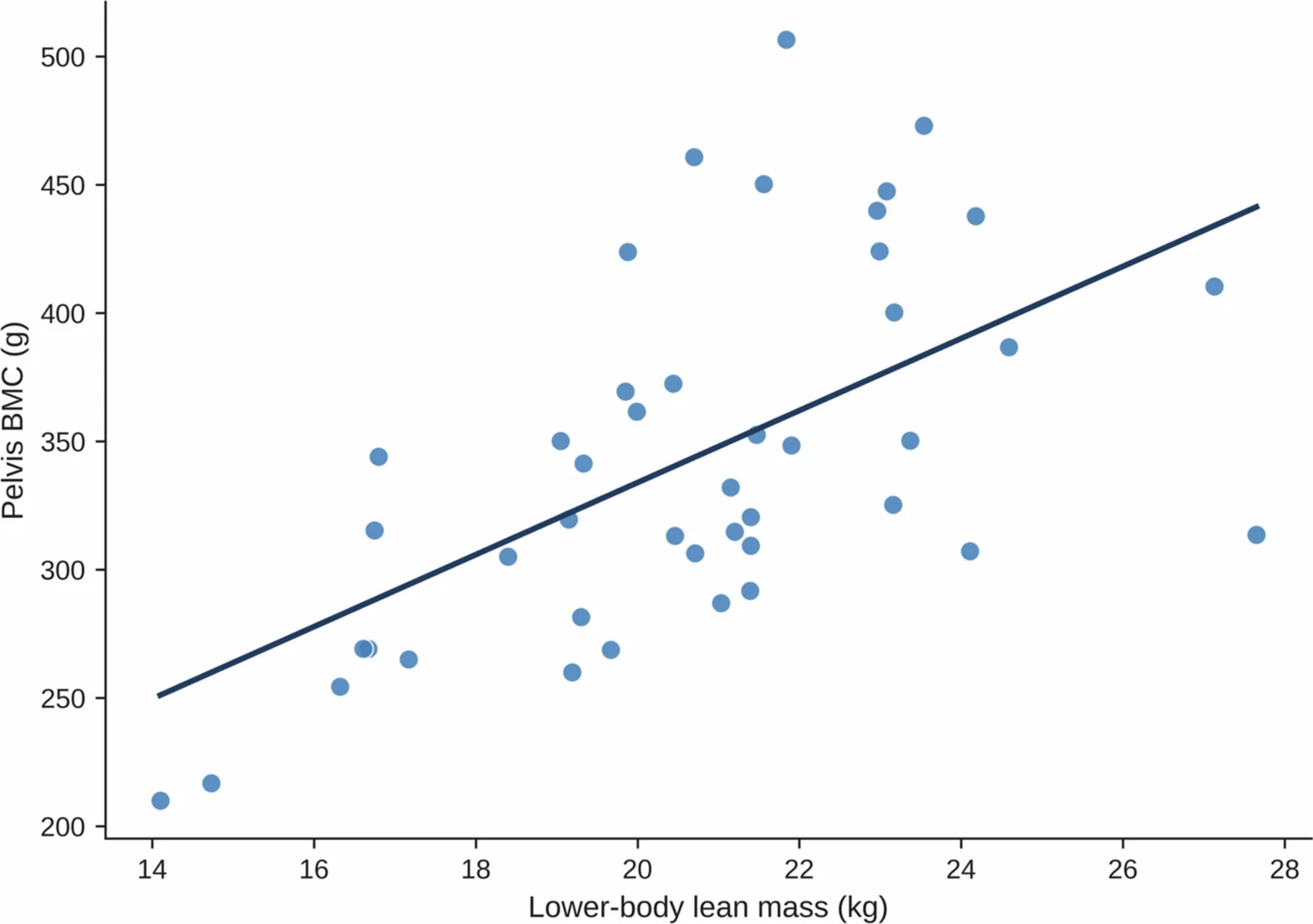
Baseline relationship between lower-body lean mass and pelvis BMC. Each point represents one of the 44 analyzed participants; randomized intervention groups are pooled because they were assigned after baseline. The solid line is the pooled descriptive ordinary least-squares fit. Pearson r = 0.586 (*P* < 0.001). Complementary body-mass, projected-area, and allometric analyzes in Table 6 show that the lean mass-pelvis BMC estimate depends materially on common size specification. BMC, bone mineral content.

### 3.3. Adjusted muscle and bone models

In models adjusted for age and height, upper body composite lean mass was associated with arm BMC (*β* = 0.605, 95% CI 0.283 to 0.928; 18 test BH q = 0.001; partial R^2^ = 0.375), arm areal BMD (*β* = 0.787, 95% CI 0.432 to 1.141; q < 0.001; partial R^2^ = 0.387), and rib BMC (*β* = 0.608, 95% CI 0.240 to 0.976; q = 0.005; partial R^2^ = 0.302). Lower body lean mass was associated with leg BMC (*β* = 0.429, 95% CI 0.080 to 0.779; q = 0.041; partial R^2^ = 0.171) and pelvis BMC (*β* = 0.550, 95% CI 0.144 to 0.956; q = 0.028; partial R^2^ = 0.180). The full conventional models are reported in Table 4 and summarized graphically in Figure 4.

**Table 4.** Conventional age- and height-adjusted cross-sectional associations between broad regional lean-mass compartments and regional bone measures.

Family	Outcome	Residual df	Standardized *β* (95% CI)	Unstandardized coefficient (95% CI)	P	Family BH q	18-test BH q	Partial R^2^
Upper-body muscle-bone	Arm BMC	40	0.605 (0.283 to 0.928)	8.99 (4.20 to 13.78)	<0.001	<0.001	0.001	0.375
	Arm areal BMD	40	0.787 (0.432 to 1.141)	0.0122 (0.0067 to 0.0178)	<0.001	<0.001	<0.001	0.387
	Rib BMC	40	0.608 (0.240 to 0.976)	4.68 (1.85 to 7.51)	0.001	0.002	0.005	0.302
	Rib areal BMD	40	0.297 (−0.155 to 0.748)	0.0048 (−0.0025 to 0.0121)	0.198	0.237	0.297	0.055
	Thoracic-spine BMC	40	0.445 (−0.017 to 0.906)	2.68 (−0.10 to 5.47)	0.059	0.088	0.118	0.141
	Thoracic-spine areal BMD	40	0.199 (−0.355 to 0.753)	0.0057 (−0.0102 to 0.0216)	0.482	0.482	0.590	0.023
Lower-body muscle-bone	Leg BMC	40	0.429 (0.080 to 0.779)	30.07 (5.59 to 54.55)	0.016	0.032	0.041	0.171
	Leg areal BMD	40	0.446 (−0.031 to 0.923)	0.0224 (−0.0016 to 0.0464)	0.067	0.089	0.121	0.094
	Pelvis BMC	40	0.550 (0.144 to 0.956)	13.19 (3.46 to 22.92)	0.008	0.032	0.028	0.180
	Pelvis areal BMD	40	0.365 (−0.101 to 0.832)	0.0172 (−0.0048 to 0.0393)	0.125	0.125	0.204	0.060

HC3 confidence intervals and *P* values are reported. These models characterize the broad DXA phenotype and are not interpreted as size-independent muscle-bone effects. The body-mass, projected-area, and allometric models in Table 6 are complementary analyzes with equal interpretive importance for evaluating common scaling. The 18-test BH q column applies Benjamini-Hochberg correction across all adjusted cross-sectional tests.

**Figure 4.**
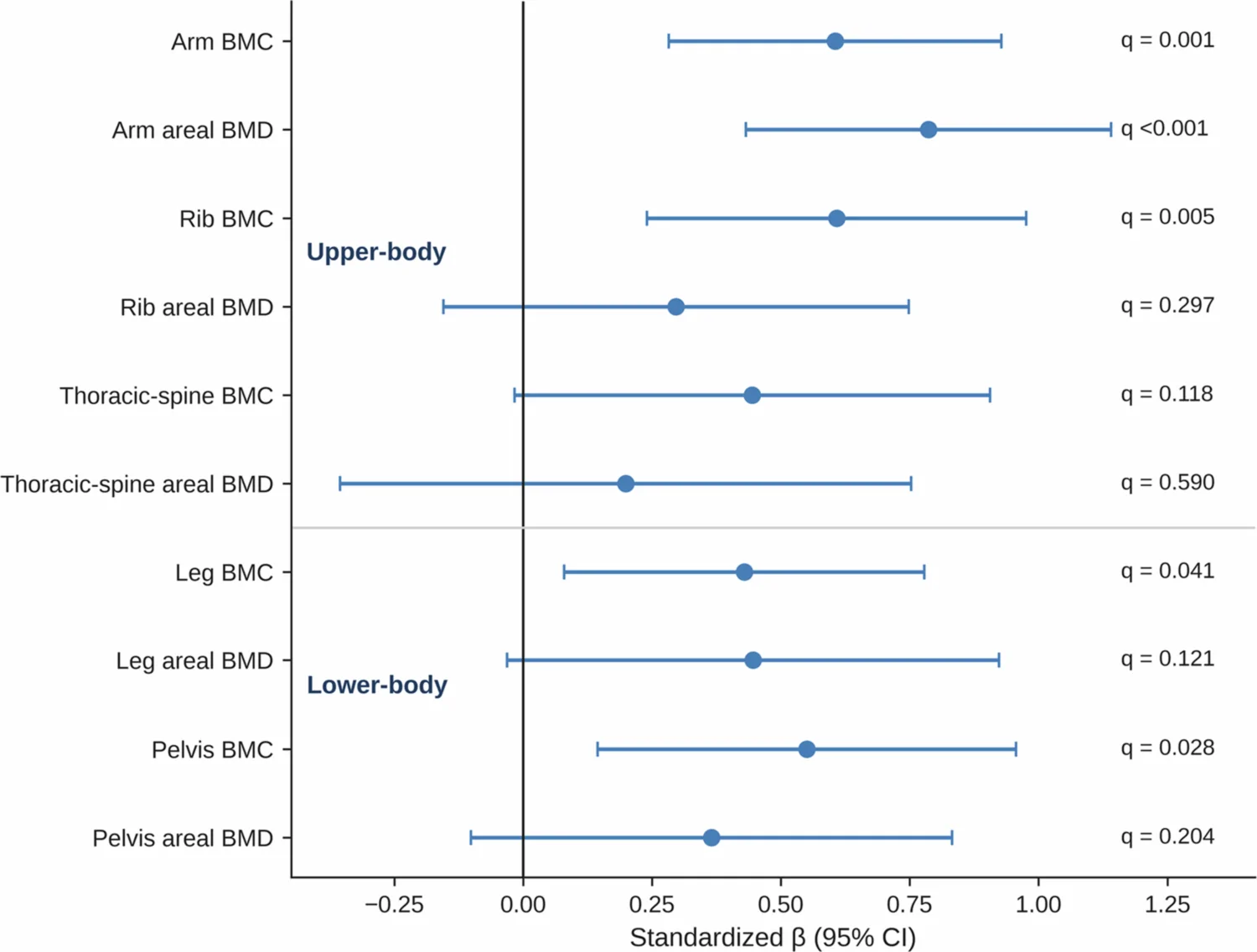
Conventional age- and height-adjusted cross-sectional regional lean mass-bone associations. Points are standardized *β* coefficients and horizontal bars are HC3 95% confidence intervals (*n* = 44). q values use Benjamini-Hochberg correction across all 18 adjusted cross-sectional tests. These conventional models describe a broad DXA phenotype and should be interpreted together with the body-mass, projected-area, and allometric analyzes in Table 6, which are equally important for evaluating common scaling. BMC, bone mineral content; BMD, DXA-derived areal bone mineral density; CI, confidence interval.

### 3.4. Bone parameters and physical function

After adjustment for age, height, and the available lower body lean mass measure, pelvis BMC was conditionally associated with estimated leg press 1-RM (*β* = 0.501, 95% CI 0.235 to 0.766; 18 test BH q = 0.001; partial R^2^ = 0.286). Leg BMC showed a smaller conditional association with estimated leg press 1-RM (*β* = 0.446, 95% CI 0.098 to 0.795; q = 0.036; partial R^2^ = 0.147), whereas the pelvis areal BMD and vertical jump estimate did not meet the 18 test threshold (Table 5). The pelvis BMC association is illustrated in Figure 5, and the corresponding leg BMC plot is provided in online resource Fig. S1.

**Table 5.** Conditional cross-sectional associations between regional bone measures and estimated strength or performance.

Family	Association	Residual df	Standardized *β* (95% CI)	Unstandardized coefficient (95% CI)	P	Family BH q	18-test BH q	Partial R^2^
Upper-body function-bone	Estimated chest press 1-RM - arm BMC	39	0.165 (−0.311 to 0.641)	0.041 (−0.076 to 0.157)	0.497	0.890	0.590	0.017
	Upper-body peak power - arm BMC	39	0.299 (−0.178 to 0.775)	0.290 (−0.173 to 0.753)	0.219	0.877	0.303	0.042
	Pull-ups - rib areal BMD	39	−0.079 (−0.460 to 0.302)	−3.0866 (−17.9924 to 11.8192)	0.685	0.890	0.725	0.006
	Estimated chest press 1-RM - thoracic-spine areal BMD	39	0.020 (−0.260 to 0.300)	2.5139 (−33.1338 to 38.1616)	0.890	0.890	0.890	0.001
Lower-body function-bone	Estimated leg press 1-RM - leg BMC	39	0.446 (0.098 to 0.795)	0.154 (0.034 to 0.274)	0.012	0.024	0.036	0.147
	Lower-body peak power - leg BMC	39	0.163 (−0.340 to 0.667)	0.061 (−0.128 to 0.250)	0.525	0.525	0.590	0.015
	Vertical-jump height - pelvis areal BMD	39	0.374 (0.034 to 0.714)	19.4684 (1.7736 to 37.1632)	0.031	0.041	0.070	0.134
	Estimated leg press 1-RM - pelvis BMC	39	0.501 (0.235 to 0.766)	0.504 (0.237 to 0.772)	<0.001	<0.001	0.001	0.286

Models adjusted for age, height, and the available broad regional lean-mass measure. These coefficients describe associations conditional on the measured covariates; they do not isolate an independent skeletal contribution or demonstrate a functional effect of bone mineral on estimated strength. Residual confounding by skeletal dimensions, body proportionality, training history, and biomechanics remains possible.

**Figure 5.**
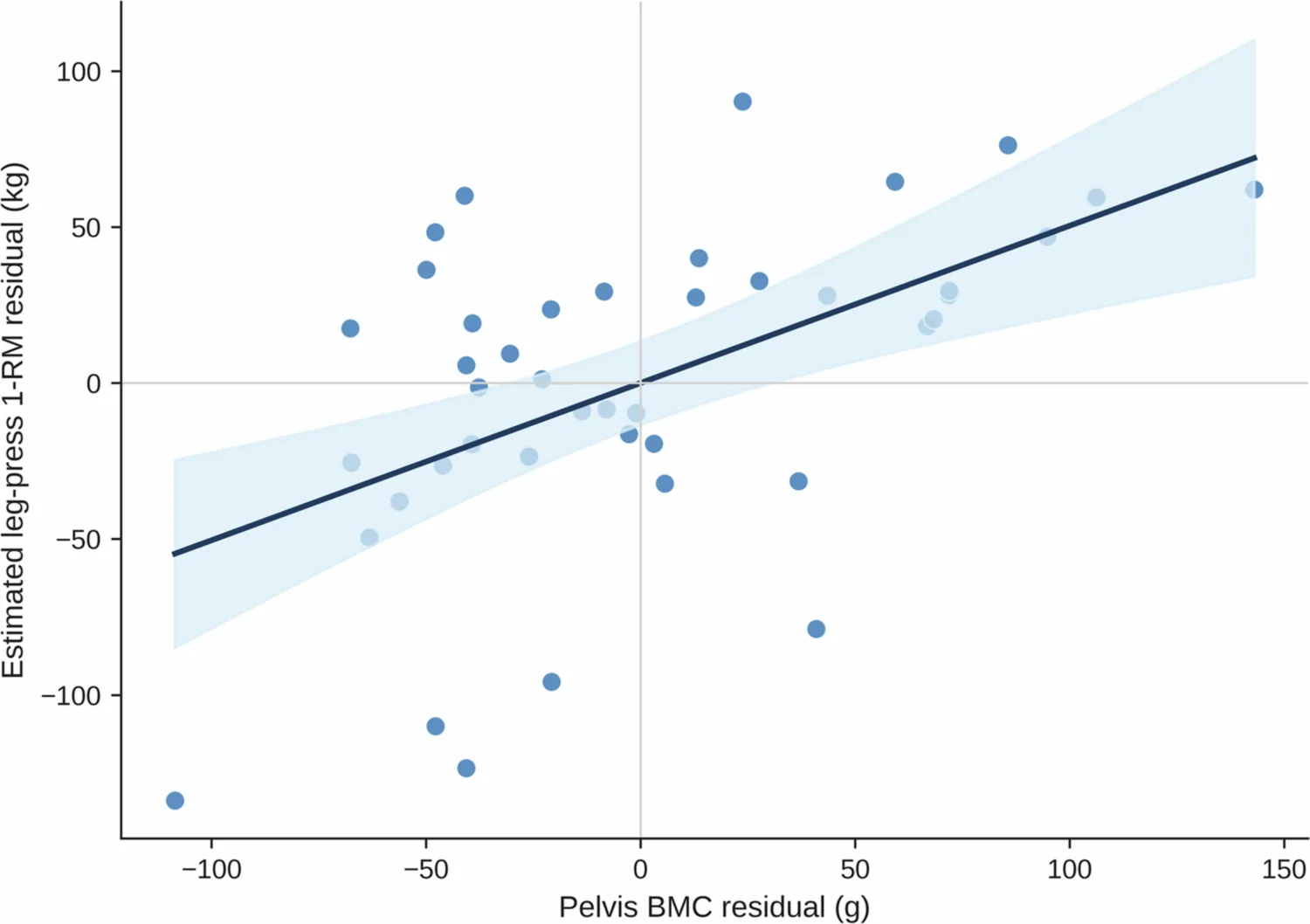
Secondary conditional baseline association between pelvis BMC and estimated leg press 1-RM. Pelvis BMC and estimated leg press 1-RM were separately residualized for age, height, and lower-body lean mass. The plot visualizes a conditional association, not an independent skeletal contribution to strength. The relationship may still reflect skeletal frame, body proportionality, cumulative loading, biomechanics, or other unmeasured characteristics. BMC, bone mineral content; 1-RM, repetition-derived estimated one-repetition maximum.

### 3.5. Exploratory longitudinal analysis (online resource)

Exploratory longitudinal models are reported only in online resource Table S4. They are not used to support the main conclusions because the study was not powered to detect small associations between individual responses and regional DXA least significant change values were unavailable.

### 3.6. Diagnostics and complementary size analyzes

The complementary size analyzes were central to the interpretation. Lower body lean mass was strongly correlated with body mass, leg projected area, and leg BMC, while pelvis BMC was strongly correlated with pelvis projected area (online resource Table S2A); the corresponding upper body scaling relationships and variance inflation diagnostics are shown in online resource Tables S2B and S2C. Models incorporating body mass, projected area, or allometric scaling weakened several relationships between lean mass and BMC. These complementary model specifications are reported in Table 6. The association between pelvis BMC and estimated leg press 1-RM remained positive in models using body mass relative strength and log log allometric scaling, but these approaches cannot remove residual confounding from skeletal dimensions, training history, or biomechanics. Detailed influence, leave one out, Huber, and bootstrap results are provided in online resource Table S3.

**Table 6.** Complementary body size, skeletal size, and allometric analyzes for the principal BMC associations.

Association	Specification	Effect scale	Estimate (95% CI)	*P*
Arm BMC - upper-body composite lean mass	Primary	Standardized beta	0.605 (0.283 to 0.928)	<0.001
	+randomized assignments	Standardized beta	0.629 (0.341 to 0.918)	<0.001
	+baseline body mass	Standardized beta	0.326 (−0.094 to 0.745)	0.128
	+projected bone area	Standardized beta	0.367 (0.156 to 0.579)	<0.001
	Allometric log-log size model	Elasticity	0.407 (−0.042 to 0.856)	0.076
Rib BMC - upper-body composite lean mass	Primary	Standardized beta	0.608 (0.240 to 0.976)	0.001
	+randomized assignments	Standardized beta	0.629 (0.275 to 0.984)	<0.001
	+baseline body mass	Standardized beta	0.382 (−0.079 to 0.844)	0.104
	+projected bone area	Standardized beta	0.211 (−0.108 to 0.530)	0.194
	Allometric log-log size model	Elasticity	0.488 (−0.071 to 1.047)	0.087
Leg BMC - lower-body lean mass	Primary	Standardized beta	0.429 (0.080 to 0.779)	0.016
	+randomized assignments	Standardized beta	0.464 (0.148 to 0.780)	0.004
	+baseline body mass	Standardized beta	0.302 (−0.170 to 0.774)	0.210
	+projected bone area	Standardized beta	0.240 (−0.045 to 0.525)	0.098
	Allometric log-log size model	Elasticity	0.410 (−0.206 to 1.025)	0.192
Pelvis BMC - lower-body lean mass	Primary	Standardized beta	0.550 (0.144 to 0.956)	0.008
	+randomized assignments	Standardized beta	0.580 (0.189 to 0.972)	0.004
	+baseline body mass	Standardized beta	0.488 (−0.317 to 1.292)	0.235
	+projected bone area	Standardized beta	0.122 (−0.157 to 0.401)	0.393
	Allometric log-log size model	Elasticity	0.873 (−0.095 to 1.842)	0.077
Estimated leg press 1-RM - leg BMC	Primary	Standardized beta	0.446 (0.098 to 0.795)	0.012
	+randomized assignments	Standardized beta	0.535 (0.091 to 0.979)	0.018
	+baseline body mass	Standardized beta	0.445 (0.102 to 0.787)	0.011
	+projected bone area	Standardized beta	0.593 (0.188 to 0.999)	0.004
	Allometric log-log size model	Elasticity	0.540 (0.152 to 0.928)	0.006
	Body-mass-relative estimated 1-RM outcome	Standardized beta	0.408 (0.046 to 0.770)	0.027
Estimated leg press 1-RM - pelvis BMC	Primary	Standardized beta	0.501 (0.235 to 0.766)	<0.001
	+randomized assignments	Standardized beta	0.538 (0.237 to 0.839)	<0.001
	+baseline body mass	Standardized beta	0.499 (0.199 to 0.799)	0.001
	+projected bone area	Standardized beta	0.376 (0.026 to 0.726)	0.035
	Allometric log-log size model	Elasticity	0.454 (0.145 to 0.763)	0.004
	Body-mass-relative estimated 1-RM outcome	Standardized beta	0.514 (0.243 to 0.785)	<0.001

These analyzes are given equal interpretive weight with the conventional age- and height-adjusted models when evaluating common scaling. The plus sign (+) indicates covariates added to the conventional model. Body-mass adjustment reduces shared overall-size variation; projected-area adjustment estimates BMC conditional on two-dimensional skeletal area; and log-log allometric models assess multiplicative scaling. None of these specifications is interpreted as identifying a unique causal skeletal effect.

## 4. Discussion

The main finding is that regional DXA lean mass and bone measures describe a phenotype that is strongly shaped by body size in resistance-trained males. Several positive associations between lean mass and BMC were evident in models adjusted for age and height, but these relationships weakened or became less precise when overall body size, projected skeletal area, or allometric scaling was considered. Pelvis BMC was also conditionally associated with estimated leg press 1-RM, although this finding is best viewed as part of the same size and proportion dependent phenotype rather than evidence of a separate bone and function mechanism.

The regional associations between muscle and bone are compatible with the mechanostat concept and with the broader view that these tissues function together [1–3]. Repeated contractions expose nearby skeletal regions to combinations of tensile, compressive, bending, and torsional loads [1,2,4]. Over years, individuals who develop or maintain more regional muscle may also accumulate greater skeletal mineral or larger skeletal dimensions at habitually loaded sites. Yet several common causes remain equally plausible. Larger individuals generally possess both more lean tissue and greater skeletal mineral quantity, while growth, genetics, endocrine exposure, diet, habitual activity, and training history can influence both tissues [5–7]. Age- and height-adjusted models therefore describe the broader trained phenotype rather than isolating a muscle-specific effect. In this manuscript, the mechanostat is used only as physiological context; the broad compartment measurements and cross-sectional design do not test mechanostat theory or a site-specific muscle-to-bone mechanism.

The size of the unadjusted regional associations was broadly comparable with earlier reports in young men, although the studies are not directly interchangeable. Guimarães and colleagues reported adjusted R^2^ values of 0.74 for upper limb lean mass with upper limb BMC and 0.68 for lower limb lean mass with lower limb BMC [7], while Sutter and colleagues reported correlations of approximately r = 0.54 to 0.64 between appendicular lean mass index and BMD at several skeletal sites [5]. In the current resistance trained sample, the corresponding unadjusted correlations were r = 0.780 for upper body composite lean mass with arm BMC and r = 0.720 for lower body lean mass with leg BMC. The broadly similar raw magnitudes are consistent with a regional phenotype, whereas the attenuation seen in our size conditioned models shows why these correlations should not be interpreted as size independent coupling. Differences in training status, regional definitions, strength tests, and statistical adjustment also limit direct quantitative comparison.

The scaling analyzes are therefore part of the main interpretation rather than simply secondary checks. Body mass, regional lean mass, projected bone area, and BMC were strongly interrelated, and the associations between lean mass and BMC became notably less precise under allometric scaling. The models adjusted for age and height should therefore be read as descriptions of a broad DXA phenotype that still contains substantial body size information, not as evidence of muscle and bone effects that are independent of size. Models that adjust BMC for projected area ask how mineral content varies at a given two-dimensional skeletal area, whereas areal BMD is calculated as BMC divided by area. These approaches are related, but they are not interchangeable.

The more consistent pattern seen for BMC also requires cautious interpretation. BMC preserves mineral quantity and projected size information within a DXA region, whereas areal BMD partially normalizes mineral content by projected area. Because DXA is a two-dimensional projection method, neither measure directly quantifies volumetric density, geometry, cortical thickness, trabecular architecture, or mechanical competence [8]. Stronger BMC relationships may reflect accumulated loading and skeletal size, but they may also arise because BMC shares more size-related variance with lean mass. BMC and areal BMD are therefore complementary descriptors rather than interchangeable measures or direct surrogates of bone strength.

The association between pelvis BMC and estimated leg press 1-RM is a secondary conditional finding. One possible biomechanical explanation is that pelvis BMC partly tracks the size and proportions of the pelvis and hip complex. Those features could covary with hip position, femoral lever arms, trunk to pelvis force transmission, and the ability to tolerate large external loads during a plate loaded leg press. Cumulative lower body loading could also contribute to both a larger pelvic mineral signal and greater estimated strength. However, DXA BMC does not measure pelvic geometry, moment arms, cortical structure, or mechanical competence, so the observed association cannot distinguish these possibilities from common scaling or training history. Lower body lean mass may itself lie on pathways linking body size, training exposure, and performance. Pelvis BMC should therefore not be interpreted as an independent determinant of strength or used as a practical surrogate for strength assessment or training prescription.

Interpretation of the upper body findings is also limited by the level of anatomical detail available. Upper body composite lean mass combined both arms with the entire trunk, yet it was compared separately with arm, rib, and thoracic spine outcomes. These are broad compartment comparisons rather than site matched muscle and bone units. The unadjusted relationship between arm BMC and estimated chest press 1-RM was largely attenuated after age, height, and upper body composite lean mass were included in the model. Because arm only and trunk specific lean mass exports were unavailable in the archived dataset, more anatomically specific upper body analyzes could not be reconstructed.

The exploratory longitudinal analysis addressed a different question over a much shorter period than the baseline cross-sectional analysis. Because the parent trial was not powered to detect small associations between individual responses and regional DXA least significant change values were unavailable, these models are retained only in the online resource and are not used to support the main conclusions.

A strength of the analysis is that it shows clearly how the interpretation changes with model specification. The main text therefore emphasizes the conventional phenotype models together with the body mass, projected area, allometric, and study wide multiplicity results. More detailed HC3 diagnostics, leave one out estimates, Huber models, bootstrap intervals, and variance inflation calculations are retained in the online resource for reproducibility rather than presented as additional layers of physiological evidence.

Several limitations should be considered when interpreting these findings. This was a secondary analysis and the hypotheses were not prospectively registered. Only the 44 completers were available in the archived participant level dataset, and baseline records for the four participants who later withdrew could not be recovered. Lean mass, projected area, BMC, and BMD came from the same DXA scan and may therefore share mathematical structure related to body size as well as correlated measurement error. Regional bone scan rescan precision, least significant change values, analyst blinding, and detailed records of manual region editing were unavailable. The upper body lean mass composite was anatomically broad. The main performance outcome was an estimated 1-RM derived from no more than 10 repetitions, and important apparatus details and test-retest reliability were unavailable. Resistance training age was reported at group level in the parent paper, but participant level cumulative loading, sport participation, habitual exercise volume, limb lengths, pelvic dimensions, technique, muscle quality, calcium and vitamin D exposure, and endocrine variables were not available in this dataset. Any of these factors could influence both BMC and estimated leg press performance. The sample also had limited power to detect smaller partial associations, and influential observations remained relevant despite the robustness analyzes. The sample consisted entirely of young adult resistance-trained males, so the DXA phenotype described here should not be assumed to apply to females, older adults, adolescents, untrained individuals, or clinical populations. Finally, because the archived baseline analysis included only participants who later completed the intervention, some selection bias is possible and cannot be quantified without the four missing baseline records. These limitations are also central to practical interpretation: the present associations do not establish a clinically or performance relevant threshold for regional BMC and do not support using DXA bone measures to estimate individual strength, judge training readiness, or prescribe resistance exercise.

Future studies should examine these questions prospectively in larger and more diverse trained samples, using direct strength testing, detailed histories of recent and lifelong loading, anthropometric and skeletal geometry measures, and imaging methods that can assess volumetric density and bone structure. Site specific DXA precision should also be established before individual regional changes are interpreted. These designs could help determine whether skeletal geometry or loading history explains the conditional association between pelvis BMC and estimated 1-RM, and whether any of these muscle and bone relationships have practical value for performance or bone health assessment.

## 5. Conclusion

Broad regional lean mass and bone measures were related at baseline in these resistance-trained males, but the main interpretation is that these relationships depend substantially on common body and skeletal scaling. Several BMC associations seen in models adjusted for age and height weakened or became imprecise when body mass, projected area, or allometric scaling was considered. Pelvis BMC was conditionally associated with estimated leg press 1-RM, but this relationship may reflect skeletal size, body proportions, loading history, or other unmeasured characteristics and should not be interpreted as an independent skeletal contribution to strength. Overall, the findings support cautious characterization of a body size sensitive DXA phenotype rather than a new muscle and bone mechanism.

## Supplementary Material

TablesF.docx TablesF.docx

TablesF.docx TablesF.docx

## Data Availability

Public deposition of participant level data remains subject to ethics and institutional data-sharing requirements; qualified researchers may also request data from the corresponding author.
