# Supplementary material for: Regional lean mass, bone mineral measures, and estimated leg press 1-RM in resistance-trained males
Source: J Int Soc Sports Nutr. 2026 Oct 11;23(1):2746457. doi: 10.1080/15502783.2026.2746457 (PMC13656645; doi:10.1080/15502783.2026.2746457)
Supplement: Supplementary file 1 [file RSSN_A_2746457_SM0966.docx]

**Online Resource Fig. S1 Conditional baseline association between bilateral leg BMC and estimated leg press 1-RM**

Bilateral leg BMC and estimated leg press 1-RM were separately residualized for age, height, and lower-body lean mass. The primary standardized estimate was β=0.446 (95% CI 0.098 to 0.795; 18-test cross-sectional BH q=0.036). Under the broader 26-test sensitivity that also includes exploratory longitudinal models, q=0.052. The plot is therefore retained as a secondary Online Resource figure. BMC, bone mineral content; 1-RM, repetition-derived estimated one-repetition maximum.
