# Supplementary material for: Regional lean mass, bone mineral measures, and estimated leg press 1-RM in resistance-trained males
Source: J Int Soc Sports Nutr. 2026 Oct 11;23(1):2746457. doi: 10.1080/15502783.2026.2746457 (PMC13656645; doi:10.1080/15502783.2026.2746457)
Supplement: Supplementary file 2 [file RSSN_A_2746457_SM0967.docx]

**Online Resource Tables**

**Online Resource Table S1. Consolidated participant flow for the parent trial and publications.**

| **Stage** | **n** | **Clarification** |
| --- | --- | --- |
| Assessed for eligibility | 112 | Parent-trial participant flow |
| Did not meet eligibility criteria | 28 | Excluded before randomization |
| Declined after initial interview | 36 | Did not enter the randomized trial |
| Randomized | 48 | 12 assigned to each of CT+1.6, CT+3.2, RT+1.6, RT+3.2 |
| Withdrew after randomization | 4 | One per group: lack of time, loss of interest, COVID-19, or musculoskeletal injury |
| Completed / final analyzed cohort | 44 | 11 per group; final participant-level analyses/figures in prior publications and current archived dataset |
| Available to current baseline reanalysis | 44 | Archived individual-level dataset did not contain the four later withdrawals; their baseline records could not be retrieved for this revision |

*The apparent 48-versus-44 discrepancy reflects randomized allocation versus final analysis. Some prior abstracts/method summaries describe the randomized groups as n=12 each, whereas participant-flow/results sections document one withdrawal per group and final analyses with n=11 each. The current analysis uses the same 44 completers, not a different cohort.*

**Online Resource Table S2A. Lower-body body-size and skeletal-size Pearson correlation matrix (n=44).**

| **Variable** | **Height** | **Body mass** | **Lower-body lean mass** | **Leg projected area** | **Leg BMC** | **Pelvis projected area** | **Pelvis BMC** | **Estimated leg press 1-RM** |
| --- | --- | --- | --- | --- | --- | --- | --- | --- |
| Height | 1.000 | 0.427 | 0.708 | 0.861 | 0.725 | 0.660 | 0.513 | -0.117 |
| Body mass | 0.427 | 1.000 | 0.753 | 0.488 | 0.547 | 0.254 | 0.403 | 0.421 |
| Lower-body lean mass | 0.708 | 0.753 | 1.000 | 0.755 | 0.720 | 0.627 | 0.586 | 0.296 |
| Leg projected area | 0.861 | 0.488 | 0.755 | 1.000 | 0.829 | 0.706 | 0.582 | 0.018 |
| Leg BMC | 0.725 | 0.547 | 0.720 | 0.829 | 1.000 | 0.725 | 0.805 | 0.233 |
| Pelvis projected area | 0.660 | 0.254 | 0.627 | 0.706 | 0.725 | 1.000 | 0.845 | 0.146 |
| Pelvis BMC | 0.513 | 0.403 | 0.586 | 0.582 | 0.805 | 0.845 | 1.000 | 0.331 |
| Estimated leg press 1-RM | -0.117 | 0.421 | 0.296 | 0.018 | 0.233 | 0.146 | 0.331 | 1.000 |

*This matrix documents the substantial common scaling among height, body mass, lower-body lean mass, projected bone area, BMC, and estimated leg press 1-RM. These correlations support treating size-conditioned analyses as part of the principal interpretation rather than as peripheral checks.*

**Online Resource Table S2B. Upper-body body-size and skeletal-size Pearson correlation matrix (n=44).**

| **Variable** | **Height** | **Body mass** | **Upper-body composite lean mass** | **Arm projected area** | **Arm BMC** | **Rib projected area** | **Rib BMC** | **Thoracic-spine projected area** | **Thoracic-spine BMC** |
| --- | --- | --- | --- | --- | --- | --- | --- | --- | --- |
| Height | 1.000 | 0.427 | 0.529 | 0.784 | 0.616 | 0.542 | 0.572 | 0.427 | 0.359 |
| Body mass | 0.427 | 1.000 | 0.820 | 0.614 | 0.760 | 0.529 | 0.617 | 0.706 | 0.605 |
| Upper-body composite lean mass | 0.529 | 0.820 | 1.000 | 0.667 | 0.780 | 0.657 | 0.663 | 0.724 | 0.569 |
| Arm projected area | 0.784 | 0.614 | 0.667 | 1.000 | 0.875 | 0.653 | 0.724 | 0.628 | 0.635 |
| Arm BMC | 0.616 | 0.760 | 0.780 | 0.875 | 1.000 | 0.640 | 0.826 | 0.662 | 0.781 |
| Rib projected area | 0.542 | 0.529 | 0.657 | 0.653 | 0.640 | 1.000 | 0.835 | 0.494 | 0.351 |
| Rib BMC | 0.572 | 0.617 | 0.663 | 0.724 | 0.826 | 0.835 | 1.000 | 0.596 | 0.653 |
| Thoracic-spine projected area | 0.427 | 0.706 | 0.724 | 0.628 | 0.662 | 0.494 | 0.596 | 1.000 | 0.706 |
| Thoracic-spine BMC | 0.359 | 0.605 | 0.569 | 0.635 | 0.781 | 0.351 | 0.653 | 0.706 | 1.000 |

*Upper-body composite lean mass includes bilateral arms and trunk. These correlations therefore describe broad compartment scaling and should not be interpreted as anatomically matched muscle-bone relationships.*

**Online Resource Table S2C. Variance-inflation diagnostics for key lower-body function models.**

| **Model** | **Maximum VIF** | **Variable with maximum VIF** |
| --- | --- | --- |
| Pelvis BMC-function primary | 2.80 | lower_body_lean_mass_pre_kg |
| Pelvis BMC-function + body mass | 4.41 | lower_body_lean_mass_pre_kg |
| Pelvis BMC-function + projected area | 5.38 | pelvis_area_pre_cm2 |
| Leg BMC-function primary | 2.94 | height_cm |
| Leg BMC-function + body mass | 4.25 | lower_body_lean_mass_pre_kg |
| Leg BMC-function + projected area | 6.19 | legs_area_pre_cm2 |

*VIF, variance-inflation factor. Collinearity increased when projected area or body mass was added, consistent with shared body-size structure.*

**Online Resource Table S3. Influence and robustness analyses for principal BMC associations.**

| **Association** | **Primary β (HC3 95% CI)** | **Leave-one-out β range** | **Huber β** | **Bootstrap 95% CI** | **Max leverage** | **Max \|studentized residual\|** | **Max Cook's D** |
| --- | --- | --- | --- | --- | --- | --- | --- |
| Arm BMC - upper-body composite lean mass | 0.605 (0.283 to 0.928) | 0.573 to 0.665 | 0.574 | 0.420 to 0.821 | 0.217 | 2.84 | 0.276 |
| Rib BMC - upper-body composite lean mass | 0.608 (0.240 to 0.976) | 0.568 to 0.704 | 0.643 | 0.355 to 0.889 | 0.217 | 2.80 | 0.271 |
| Leg BMC - lower-body lean mass | 0.429 (0.080 to 0.779) | 0.353 to 0.511 | 0.442 | 0.123 to 0.739 | 0.236 | 2.24 | 0.157 |
| Pelvis BMC - lower-body lean mass | 0.550 (0.144 to 0.956) | 0.471 to 0.652 | 0.578 | 0.162 to 0.870 | 0.236 | 2.77 | 0.272 |
| Estimated leg press 1-RM - leg BMC | 0.446 (0.098 to 0.795) | 0.342 to 0.511 | 0.407 | 0.124 to 0.767 | 0.240 | 2.66 | 0.372 |
| Estimated leg press 1-RM - pelvis BMC | 0.501 (0.235 to 0.766) | 0.409 to 0.546 | 0.460 | 0.232 to 0.728 | 0.285 | 2.48 | 0.333 |

*Detailed influence, leave-one-out, Huber, and bootstrap results are retained here for reproducibility but are no longer emphasized as separate inferential layers in the main manuscript. They assess coefficient stability and cannot remove unmeasured confounding, common body-size structure, or correlated DXA measurement error.*

**Online Resource Table S4. Exploratory longitudinal models of regional lean-mass change and post-intervention bone outcomes.**

| **Family** | **Outcome** | **Residual df** | **Standardized β (95% CI)** | **P** | **Family BH q** | **Partial R²** |
| --- | --- | --- | --- | --- | --- | --- |
| Upper-body longitudinal | Post arm BMC | 38 | 0.012 (-0.053 to 0.077) | 0.712 | 0.712 | 0.003 |
|  | Post arm areal BMD | 38 | 0.025 (-0.084 to 0.134) | 0.653 | 0.712 | 0.005 |
|  | Post rib BMC | 38 | 0.090 (-0.039 to 0.220) | 0.172 | 0.689 | 0.041 |
|  | Post rib areal BMD | 38 | -0.039 (-0.192 to 0.115) | 0.620 | 0.712 | 0.007 |
| Lower-body longitudinal | Post leg BMC | 38 | 0.027 (-0.066 to 0.119) | 0.571 | 0.571 | 0.008 |
|  | Post leg areal BMD | 38 | -0.055 (-0.169 to 0.058) | 0.339 | 0.571 | 0.019 |
|  | Post pelvis BMC | 38 | -0.100 (-0.223 to 0.023) | 0.110 | 0.440 | 0.096 |
|  | Post pelvis areal BMD | 38 | -0.061 (-0.221 to 0.098) | 0.453 | 0.571 | 0.019 |

*These longitudinal models are exploratory and are retained only for completeness. The parent trial was not powered for small individual-response associations, regional DXA least-significant-change values were unavailable, and the pooled four-group analysis cannot establish homogeneous response coupling. They are not used to support the main conclusions.*

**Online Resource Table S5. Conservative Benjamini-Hochberg sensitivity across all 26 adjusted cross-sectional and exploratory longitudinal tests.**

| **Family** | **Outcome** | **Standardized β (95% CI)** | **P** | **BH q across all 26** |
| --- | --- | --- | --- | --- |
| Upper-body muscle-bone | Arm BMC | 0.605 (0.283 to 0.928) | <0.001 | 0.002 |
|  | Arm areal BMD | 0.787 (0.432 to 1.141) | <0.001 | <0.001 |
|  | Rib BMC | 0.608 (0.240 to 0.976) | 0.001 | 0.008 |
|  | Rib areal BMD | 0.297 (-0.155 to 0.748) | 0.198 | 0.367 |
|  | Thoracic-spine BMC | 0.445 (-0.017 to 0.906) | 0.059 | 0.170 |
|  | Thoracic-spine areal BMD | 0.199 (-0.355 to 0.753) | 0.482 | 0.680 |
| Lower-body muscle-bone | Leg BMC | 0.429 (0.080 to 0.779) | 0.016 | 0.060 |
|  | Leg areal BMD | 0.446 (-0.031 to 0.923) | 0.067 | 0.174 |
|  | Pelvis BMC | 0.550 (0.144 to 0.956) | 0.008 | 0.041 |
|  | Pelvis areal BMD | 0.365 (-0.101 to 0.832) | 0.125 | 0.271 |
| Upper-body function-bone | Estimated chest press 1-RM - arm BMC | 0.165 (-0.311 to 0.641) | 0.497 | 0.680 |
|  | Upper-body peak power - arm BMC | 0.299 (-0.178 to 0.775) | 0.219 | 0.380 |
|  | Pull-ups - rib areal BMD | -0.079 (-0.460 to 0.302) | 0.685 | 0.741 |
|  | Estimated chest press 1-RM - thoracic-spine areal BMD | 0.020 (-0.260 to 0.300) | 0.890 | 0.890 |
| Lower-body function-bone | Estimated leg press 1-RM - leg BMC | 0.446 (0.098 to 0.795) | 0.012 | 0.052 |
|  | Lower-body peak power - leg BMC | 0.163 (-0.340 to 0.667) | 0.525 | 0.682 |
|  | Vertical-jump height - pelvis areal BMD | 0.374 (0.034 to 0.714) | 0.031 | 0.101 |
|  | Estimated leg press 1-RM - pelvis BMC | 0.501 (0.235 to 0.766) | <0.001 | 0.002 |
| Upper-body longitudinal | Post arm BMC | 0.012 (-0.053 to 0.077) | 0.712 | 0.741 |
|  | Post arm areal BMD | 0.025 (-0.084 to 0.134) | 0.653 | 0.738 |
|  | Post rib BMC | 0.090 (-0.039 to 0.220) | 0.172 | 0.345 |
|  | Post rib areal BMD | -0.039 (-0.192 to 0.115) | 0.620 | 0.733 |
| Lower-body longitudinal | Post leg BMC | 0.027 (-0.066 to 0.119) | 0.571 | 0.707 |
|  | Post leg areal BMD | -0.055 (-0.169 to 0.058) | 0.339 | 0.551 |
|  | Post pelvis BMC | -0.100 (-0.223 to 0.023) | 0.110 | 0.260 |
|  | Post pelvis areal BMD | -0.061 (-0.221 to 0.098) | 0.453 | 0.680 |

*The primary revised multiplicity interpretation applies BH correction across the 18 adjusted cross-sectional tests because the longitudinal models are explicitly exploratory. This 26-test analysis is shown as an additional conservative sensitivity check that treats every adjusted cross-sectional and longitudinal model as one multiplicity universe.*
